# Prevalence of Giardiasis in Children Attending Semi-urban Daycare Centres in Guatemala and Comparison of 3 Giardia Detection Tests

**DOI:** 10.3329/jhpn.v31i2.16394

**Published:** 2013-06

**Authors:** Terri-Lynn Duffy, Gabriela Montenegro-Bethancourt, Noel W. Solomons, Miodrag Belosevic, M. Thomas Clandinin

**Affiliations:** ^1^Department of Agriculture, Food and Nutritional Science, University of Alberta, Canada;; ^2^Center for Studies of Sensory Impairment, Aging, and Metabolism, Guatemala;; ^3^Department of Biological Sciences, University of Alberta, Canada

**Keywords:** Child malnutrition, Diagnosis, Epidemiology, Giardiasis, Guatemala

## Abstract

*Giardia intestinalis* is an intestinal parasite widely prevalent in children attending daycare centres worldwide and has been associated with undernutrition. Stool samples from 48 Guatemalan children (aged 1.5-7 years) attending participating daycare centres were analyzed over five weeks for presence of *Giardia intestinalis* using light microscopy, ELISA, and rapid dipstick test. *Giardia* prevalence rates were 43.7% at Week 0 and 44.7% at Week 4, based on ELISA. Intensity, but not prevalence, of infection showed **a** trend toward decreased weight-for-age (1-tailed p=0.08). We believe that ELISA analysis of stool samples may be further adapted for measuring the intensity of infection in humans.

## INTRODUCTION

*Giardia intestinalis* is an intestinal protozoan parasite widely prevalent in children aged less than 5 years but the long-term health consequences of giardiasis in children have not been fully elucidated (1,2). Giardiasis has been associated with lower serum zinc, iron, and vitamin A in children, despite similar anthropometric indicators among infected and non-infected individuals (3,4).

Giardiasis can be diagnosed through identification of cysts, trophozoites and *Giardia*-specific antigens in faecal samples. Several faecal tests are available. Comparison of faecal diagnostic methods is problematic due to the lack of a true gold standard reference method (5). The ovum and parasite (O&P) method is the currently-accepted gold standard method despite its inferior sensitivity compared to enzyme-linked immunosorbent assays (ELISA), particularly when only a single faecal sample is available for analysis (6).

The factor of intensity of *Giardia* infection, often not measured or reported, may have been a confounding factor in previous studies that have failed to identify a negative impact of *Giardia* infection on the anthropometric indicators of children. Intensity of infection (cysts/g of faeces) can be determined through light microscopy by cyst concentration procedures, followed by quantification using a haemocytometer. Alternatively, the antigen concentration as determined by spectrophotometer readings of an ELISA may offer a more standardized method of assessing intensity of infection.

The main objective of this study was to determine the prevalence of asymptomatic giardiasis in children attending daycare centres near Quetzaltenango, Guatemala, over a 5-week longitudinal component that allowed for detection of new infection, self-clearance, persistent infection, or no infection. The second objective was to compare the detection and quantification of the intensity of *G. intestinalis* infection, using light microscopy, ELISA, and a rapid dipstick test.

## MATERIALS AND METHODS

Two stool samples per week from each subject were collected during Week 0 and Week 4 (i.e. a total of 4 samples per subject). Collection-kits were given to each parent on the day prior to the collection date, and instruction on how to use the kits was provided. Parents brought back the filled container the following morning, and the study staff transported those faecal samples to the nearby laboratory. Fresh faecal samples were analyzed for the presence of cysts in the faeces by microscopy, using the sucrose gradient concentration method and after staining with Lugol's solution (7). Intensity of *Giardia* infection was expressed as the number of cysts per gramme of faeces. A standardized scoop of faecal material was measured, and mass was recorded in grammes up to 2 decimals. Cysts were quantified using a haemocytometer (22 mm × 22 mm cover-slips, thickness 1) and a bright field microscope with high-power (40x) objective (7). Split faecal samples were also stored at −20 ^0^C and analyzed within 6 weeks for presence of *G. intestinalis* antigens in the faeces, using ELISA, or the dipstick test. The ProSpecT-Giardia-EZ-microplate assay (Oxoid, Cambridge, UK) was used in analyzing all faecal samples according to the manufacturer's instructions. Absorbance values were read using the co-analyzer 303+ with filters of 450 and 630 nm (Corporación Analíticos, S.A., Guatemala City, Guatemala). The absorbance values from the ELISA ranged from 0.0 to a maximum of 3.0. A value ≥0.05 was considered positive, meaning *Giardia*-specific antigen 65 was detected in the sample. RidaQuick Giardia dipstick test (R-Biopharm AG, Damstadt, Germany) was employed. The test was read as positive or negative (with a control indicator on the dipstick) within 5 minutes.

### Ethics

The Human Research Ethics Review Board of the University of Alberta and the ZUGUEME Ethics Board of Guatemala City approved the study.

### Statistical analysis

Information on height (to the nearest 0.5 cm) and weight (to the nearest 0.1 kg) for each subject was available at the time of the study. Data were analyzed using the Centers for Disease Control and Prevention (CDC) 2000 child growth standards to calculate z-scores of weight-for-height (WHZ), height-for-age (HAZ), and weight-for-age (WAZ) (8). Among the ELISA-positive subjects, the Spearman correlation value was calculated, with the absorbance values as the x-axis variables and the WAZ, HAZ, and WHZ values on the y-axis. One ELISA absorbance value per individual was used; if two positive samples were provided, the mean of both samples was used in the regression.

To compare the intensity of infection, the Spearman correlation value was determined between the number of cysts/g of faeces (based on microscopic analysis, positive samples only, n=15) and the corresponding ELISA absorbance values. Statistical analysis was performed using GraphPad Prism (version 5.03) for Windows (GraphPad Software, San Diego, California, USA, www.graphpad.com).

## RESULTS

Of the 53 children enrolled (aged 1.5-7 years), 48 children each provided at least 1 stool sample; 15 children provided at least one stool sample at both time-points and 23 children provided all 4 stool samples. All samples were analyzed by ELISA but only a subset of fresh samples (n=75) were analyzed by a single researcher through microscope due to time limitations. The corresponding 75 samples were assessed by dipstick. Based on ELISA results, 43.8% of the subjects tested positive for giardial coproantigen at Week 0. A similar prevalence (44.7%) was found at Week 4. The percentage of individuals with new infection, self-clearance, chronic infection or with no *G. intestinalis* infection is illustrated in Table 1 both for the cohort of children who each gave at least 1 stool sample at each end of the study (n=15) and for the 4-stool cohort (n=23), providing all 4 requisite stool samples. The 4-stool cohort appeared to exhibit a higher percentage of persisting infection compared to the subjects who provided 2 or 3 stool samples.

**Table 1.** Percentage of sample subjects displaying new infection, self-clearance, persistent infection, and no infection with G. *intestinalis* over a 5-week period

Infection status	^*^4-stool cohort (n=23) % (n)	^†^ Remaining subjects (n=15) % (n)
New infection	8.7 (2)	26.7 (4)
Self-clearance	8.7 (2)	13.3 (2)
Persistent infection	34.8 (8)	20.0 (3)
No infection	47.8 (11)	40.0 (6)

^*^Subjects who submitted all 4 stool samples (n=23).

^†^All remaining subjects who submitted at least 1 stool sample at each time-point (n=15)

Diagnostic agreement among the 75 samples evaluated by all three detection methods was 74.7%. Using ELISA as the gold standard, the microscopic analysis had a slightly higher specificity and positive predictive value (PPV), indicating greater confidence in positive test results relative to the dipstick method (Table 2). The dipstick method had a higher sensitivity and negative predictive value (NPV), indicating greater confidence in negative test results relative to microscopic analysis.

**Table 2.** Sensitivity, specificity, PPV^*^, and NPV^†^ of microscope and dipstick test for *Giardia* detection in stool samples, using ELISA as gold standard

Parameter	Microscope (n=75)	Dipstick (n=75)
Sensitivity	53.6	60.7
Specificity	100.0	97.9
PPV	100.0	94.4
NPV	78.3	80.7

^*^PPV=Positive predictive value;

^†^NPV=Negative predictive value

Comparison of intensity of *Giardia* infection, using the microscopic cyst counts (positive samples only, n=15) vs ELISA absorbance values indicates that all of the positive samples detected by microscopy had high ELISA absorbance values (>1.7), the majority (n=14) having near-maximal absorbance values of >2.9). Alternatively, not all of the samples with high ELISA absorbance values (>1.7) were positive for *G. intestinalis* with microscopic examination. The range in cyst concentration detected by microscope was 2.000-358.750 × 10 000 cysts/g of faeces. The Spearman correlation, using scaling values as proxies for intensity of infection between cyst count and absorbance reading, was non-significant (p=0.26). The detection limit of the dipstick was 2.000 × 10 000 cysts/g and an ELISA OD value of 2.5.

Subjects with a higher ELISA coproantigen intensity, though not significant, tended to have lower WAZ (r=-0.32, 1-tailed p=0.08). The 1-tailed test was performed after the general consistency of a negative coefficient correlation, i.e. an inverse association between intensity of infection and growth was shown across the board for all three indicators (WAZ, WHZ, and HAZ). No significant correlations were found between ELISA coproantigen intensity and HAZ (p=0.12), or WHZ (p=0.36).

## DISCUSSION

The prevalence of giardiasis was high in the subjects examined in this study. Our findings are consistent with the literature, in which the higher detection of giardiasis was reported when a second sample was provided (9). The second stool sample detected an additional 7 cases of giardiasis. It is possible that there were undetected cases of persistent infection when only a single stool sample per week was collected. The reported prevalence rate may, therefore, be a conservative estimate.

In comparison with the microscopic and dipstick methods, the ELISA method detected almost twice as many cases of giardiasis (Table 3). The reported sensitivity of different microscopic methods for single stool analysis has ranged between 65% and 75% (9,10). The sensitivity of microscopy in our study is lower at 53.6%. The sensitivity of RidaQuick dipsticks reported in the literature is 80%, with specificity of 99.4% (10). The sensitivity in our study is lower (60.7%), with specificity comparable (97.9%) to what has been reported in the other literature (5). The lower sensitivities (microscopy and dipstick) observed in our study could be due to low faecal cyst concentrations. Asymptomatic carriers were found to have lower cyst counts (11) compared to symptomatic cases. Dipsticks consistently detected cases having the highest intensity of infection and could be useful for rapid screening and remote fieldwork settings.

**Table 3.** Summary of *Giardia* detection in faecal samples from ELISA, dipstick and microscopic analysis

Nature of samples	ELISA (n=75)	Dipstick (n=75)	Microscope (n=75)
Number of positive samples	28	18	15
Number of negative samples	47	57	60

Previous studies have found associations between ELISA intensity values and *Giardia* cyst concentration (12,13). In this study, there was no significant correlation between cyst count using microscopic analysis and ELISA OD values. This could be due to a lack of spread in the ELISA absorbance values; 93% of the 15 samples had an absorbance value of 2.9 to 3.0, indicating maximal or near-maximal absorbance. A limitation of using ELISA as a quantitative method is the maximal absorbance level of 3.0. This level is likely too low; a higher ceiling would be necessary for the cases of giardiasis with a higher intensity of infection. Further systematic dilution of samples could provide adjusted absorbance readings above the current 3.0 limit. Standardized test to determine the correlation between expanded ELISA OD and the full range of faecal cyst counts would be the next opportunity to be pursued.

## ACKNOWLEDGEMENTS

For their technical guidance and assistance, we extend our thanks to: Lic. Gloria Hidalgo Rivas, MSc student (biological laboratory La Democracia, Quetzaltenango, Guatemala); Dr. Al Shostak (Department of Biological Sciences, University of Alberta); Joy Nolte, MSc student (Boston University); Dr. Maria Jose Rios (CeSSIAM); Claudia Arriaga Godoy (CeSSIAM); and Lic. Lillian de la Cruz (Regional Supervisor, Secretaria de las Obras Sociales de la Esposa del Presidente, Guatemala).
